# Hypertonic thermal solutions as a putative strategy for respiratory biofilm disruption

**DOI:** 10.1515/med-2026-1426

**Published:** 2026-05-14

**Authors:** Marilina Falcone, Laura Pietrangelo, Antonio Guarnieri, Natasha Brancazio, Noemi Venditti, Maria Di Naro, Giulio Petronio Petronio, Roberto Di Marco, Attilio Varricchio

**Affiliations:** Department of Medicina e Scienze della Salute “V. Tiberio”, Università degli Studi del Molise, Campobasso, Molise, Italy; Natival S.r.l., Campobasso, Molise, Italy; Responsible Research Hospital, UO Laboratorio Analisi, Campobasso, Molise, Italy; Consorzio Interuniversitario per l’Ingegneria e la Medicina (CoIIM), Campobasso, Molise, Italy; Department of Drug and Health Sciences, Università degli Studi di Catania, Catania, Sicily, Italy

**Keywords:** respiratory tract infections, biofilms, hypertonic solution, thermal water

## Abstract

**Objectives:**

Upper respiratory tract infections pose a significant challenge for healthcare systems worldwide. Forming biofilms-complex polymicrobial communities encased in an exopolymer matrix plays a pivotal role in the persistence of these infections, contributing to enhanced resistance against conventional treatments and host immune responses. Consequently, developing innovative strategies to disrupt biofilms has become a therapeutic imperative.This study investigated the *in vitro* efficacy of a hypertonic solution of salsobromoiodic thermal water, in mitigating biofilm formation by key upper respiratory tract pathogens.

**Methods:**

Biofilm formation by *Staphylococcus aureus*, *Moraxella catarrhalis*, *Haemophilus influenzae*, *Streptococcus pneumoniae*, *Candida albicans*, *Candida parapsilosis*, and *Candida tropicalis* was assessed using the MBEC Assay^®^ system. The effects of the hypertonic salsobromoiodic thermal water solution were compared with standard saline (0.9 % NaCl).

**Results:**

A significant, concentration-dependent reduction in biofilm biomass was observed in all tested microorganisms when treated with the hypertonic solution. This effect was also evident in polymicrobial biofilms, indicating a broad and consistent antibiofilm potential.

**Conclusions:**

The hypertonic solution of salsobromoiodic thermal water demonstrated notable *in vitro* activity against biofilm formation by major upper respiratory tract pathogens. These findings highlight its potential as novel therapeutic approach for managing biofilm-associated respiratory infections and enhancing conventional antimicrobial treatments effectiveness.

## Introduction

A biofilm is an assemblage of microorganisms that aggregate and adhere irreversibly to biotic or abiotic surfaces, encapsulating themselves in a self-produced matrix of extracellular polymeric substances (EPS) [1], [2], [3], [4]. This multilayer structure induces profound phenotypic and genotypic changes compared to their planktonic cells, including altered growth rates and gene expression profiles [5], 6]. These adaptations enhance microbial resistance to environmental conditions, host immune defences, and antimicrobial agents [7], [8], [9].

The EPS matrix acts as a multifunctional barrier: it limits the diffusion of antibiotics and immune effectors, sequesters antimicrobial compounds, and generates nutrient and oxygen gradients that support the survival of slow-growing, stress-tolerant subpopulations [2], [3], [4, 8], 9]. Additionally, biofilm-associated microorganisms exhibit upregulation of genes related to EPS production, efflux pumps, stress responses, quorum sensing, and metabolic shifts toward persistence and dormancy [1], 6].

Biofilm formation in the nasal cavity is a well-documented phenomenon, involving a wide variety of bacteria [10]. Several species, such as *Staphylococcus aureus*, *Streptococcus pneumoniae*, *Moraxella catarrhalis*, and *Haemophilus influenzae*, have been implicated in acute and chronic rhinosinusitis (ARS and CRS) [10], [11], [12], [13], [14], [15], [16], [17]. In addition to bacteria, yeasts like *Candida* spp., and even moulds are capable of forming or contributing to biofilms in this environment, suggesting a mutualistic or synergistic role in pathogenesis [14], 15], 18].

Notably, dual-species biofilms formed by *C.* spp. and bacterial pathogens, especially *S. aureus*, have been reported in the nasal cavity. These interkingdom interactions reinforce the biofilm structure, promote metabolic cooperation and signalling, and increase antimicrobial tolerance and immune evasion [19], [20], [21]. Such interactions are increasingly recognized as a key factor in the persistence of infection and therapeutic failure, particularly in CRS [18].

Based on these observations, the microbial strains selected for this study include clinically relevant bacteria and yeasts commonly associated with rhinosinusitis and biofilm formation in the upper airways. These include *S. aureus*, *S. pneumoniae*, *M. catarrhalis*, and *H. influenzae*, along with opportunistic yeasts *Candida albicans*, *Candida parapsilosis*, and *Candida tropicalis*, which are frequently found in CRS patients, especially those with risk factors such as corticosteroid use or immunosuppression [22], [23], [24].

Moreover, the EPS matrix restricts antibiotic diffusion through a combination of physical obstruction (due to the dense polymeric network) and chemical interactions that can bind or inactivate certain drugs [25], [26], [27]. As a result, biofilm-residing bacteria may require antibiotic concentrations that are 10- to 1,000-fold higher than those needed to eliminate planktonic cells, contributing to antimicrobial resistance (AMR) [27], 28].

AMR is considered a significant threat to the public health systems in developing countries and worldwide. Infection with AMR leads to serious illnesses and prolonged hospital admissions, increases in healthcare costs, higher costs of second-line drugs, and treatment failures. In Europe and the United States of America (USA) alone, AMR has claimed over 50,000 lives each year, with thousands more dying in other countries around the globe [28]. It has been estimated that AMR has been correlated with more than nine billion euros per year just in Europe [29]. Other mechanisms contributing to AMR have also been identified, such as the development of microenvironments that promote the exchange of genes responsible for resistance, slowed growth, activation of stress responses, and microbial coordination through quorum sensing [11], [29], [30], [31].

Given biofilm-related infections significant clinical and economic impact, researchers are actively exploring innovative strategies to counteract infections resulting from biofilm proliferation [31].

A promising non-pharmacological approach consists of using hypertonic solutions enriched with minerals and trace elements naturally present in salsobromoiodic thermal waters – a term referring to mineral water rich in sodium chloride (salso-), bromide (bromo-), and iodide (iodic) ions, typically sourced from deep geothermal aquifers. These waters are classified as thermal when they emerge from springs at temperatures ≥20–25 °C [32].

The solution used in this study is considered hypertonic because its total ionic content (e.g., sodium ∼9,376 mg/L, chloride ∼15,094 mg/L, see Table 1) results in an osmolarity significantly higher than that of intracellular fluid.

**Table 1:** Physicochemical composition of Rino Term^®^ 2.5 % hypertonic salsobromoiodic thermal water solution, as certified by the manufacturer (ADL Farmaceutici S.r.l.) on the final product formulation.

Parameters	Results	Units
T° at source	51.5	°C
pH	7.26	of pH
Specific conductivity (at 20 °C)	55,080	µS/cm
Fixed residue (at 180 °C)	27,018	mg/L
Total hardness	305.1	°fH
Ammonia nitrogen (NH_4_^+^)	0.10	mg/L
Nitrous nitrogen (NO_2_^−^)	0.020 ± 0.002	mg/L
Nitric nitrogen (NO_3_^−^)	55.9 ± 5.6	mg/L
Chloride (Cl^−^)	15,094	mg/L
Sulfate (SO_4_^−^)	5	mg/L
Bicarbonate (HCO_3_^−^)	122.0	mg/L
Calcium	683	mg/L
Magnesium	327	mg/L
Sodium	9,376	mg/L
Potassium	41.4	mg/L
Dissolved iron	0.540	mg/L
Silica (SiO_2_)	15.0	mg/L
Sulfidimetric degree (H_2_S)	<0.1	mg/L
Iodide	77.5	mg/L
Bromide	143.0	mg/L
Boron	55.6	mg/L

While historically administered as thermal vapour (aerosolized water containing dissolved salts and volatile components), in our experiments the solution was applied at room temperature. Thus, the observed antibiofilm effect is attributable primarily to its mineral composition rather than thermal activation [33].

Several clinical studies, including randomized controlled trials, have demonstrated that nasal irrigation and inhalation with thermal waters – particularly those containing sulfur, bromide, iodide, and saline components – can significantly enhance mucociliary clearance, reduce neutrophilic and eosinophilic inflammation, and improve sinonasal symptoms in patients with CRS [34], [35], [36]. These effects have been observed both in allergic and non-allergic forms and are associated with improved nasal patency, reduced mucosal edema, and better quality of life scores. These formulations are particularly beneficial in promoting nasal patency and alleviating symptoms associated with CRS, with a positive impact on patients’ quality of life [34], [35], [36], [37], [38], [39], [40]. Further-more, several studies have also described the *in vitro* effects of thermal waters, including their antioxidant and anti-elastase properties [41], [42], [43], [44].

Despite these findings, the potential effects of these solutions on the microbiota and their role in biofilm modulation remain underexplored [42]. Therefore, the objective of this study was to investigate the potential benefits of a hypertonic salsobromoiodic thermal water solution in reducing biofilm formation produced by respiratory pathogens, including *S. aureus*, *M. catarrhalis*, *H. influenzae*, *S. pneumoniae*, *C. albicans*, *C. parapsilosis*, *C. tropicalis*, and a mixed culture containing all of the aforementioned bacteria and yeasts.

## Methods

### Chemicals and reagents

Columbia blood agar/broth, Sabouraud agar, YPD broth, and *Haemophilus* medium were purchased from Thermo Fisher Scientific© (Waltham, Massachusetts, USA) and prepared according to manufacturer instructions.

2 % Crystal violet solution and Glacial Acetic Acid (≥99.85 %) were purchased from Sigma-Aldrich© (St. Louis, Missouri, USA).

The Rino Term 2.5 % hypertonic solution salsobromoiodic thermal water was kindly provided by ADL Farmaceutici S.r.l., Milano, MI, Italy (Table 1).

### Bacterial and yeast strains

*S. aureus* DSM 20231, *S. aureus* DSM 21705, *M. catarrhalis* DSM 9143, *M. catarrhalis* DSM 11994, *H. influenzae* DSM 10001, *S. pneumoniae* DSM 14377, *C. albicans* DSM 1386, *C. parapsilosis* DSM 4237 and *C. tropicalis* DSM 4238. All strains used in this study were obtained from the Leibniz Institute DSMZ – German Collection of Microorganisms and Cell Cultures GmbH (https://www.dsmz.de/) and were handled according to the supplier’s recommendations.

### Bacterial and yeast growth conditions

*S. aureus*, *M. catarrhalis*, and *S. pneumoniae* were grown in Columbia medium, and incubation was carried out at 37 °C for 24 h under aerobic conditions.

*H. influenzae* was grown in *Haemophilus* Test Medium Base at 37 °C for 24 h in 5 %CO_2_.

The *Candida* strains were grown in Sabouraud agar at 30 °C for 48 h under aerobic conditions.

Each microbial strain was maintained as a monoculture until the exponential growth phase was reached. Subsequently, the cultures were diluted to a standard con-centration of 0.5McFarland, corresponding to 1.5 × 10^8^ CFU/mL for bacterial species and 1 × 10^6^ CFU/mL for fungal species.

For the polymicrobial biofilm condition, microbial suspensions of all bacterial and yeast strains used in the study (Section 2.2) were adjusted to 0.5 McFarland and combined in equal volumes to create a composite inoculum, resulting in a standardized 1:1 ratio for each strain in accordance with MBEC procedural guidelines (Innovotech, MBEC Manual v2.1). Columbia Broth was selected as the common growth medium, as it supports both bacterial and yeast species under co-culture conditions. This approach was adopted to ensure experimental reproducibility across replicates rather than to mimic clinical species abundance.

### Minimun biofilm eradication concentration (MBEC) assay

The effect of hypertonic solution salsobromoiodic thermal water in reducing biofilm formation was evaluated using the MBEC™ Biofilm Inoculator high-throughput screening system (Innovotech Inc., Edmonton, Canada) with 96-well base pegs. Biofilms were established on the pegs (108.9 mm^2^/peg) of the MBEC™ by following the manufacturer’s instructions and adopting the protocol described by ASTM with modifications [45]. 150 µL of each strain was inoculated into 96 wells MBEC plate along with negative controls (CTRL-). For each strain, 3 biological replicates (i.e., independent cultures prepared from separate inocula) and 8 technical replicates (i.e., repeated measurements from the same inoculum) were performed. Plates were incubated at 37 °C for 48 h under controlled humidity conditions to promote biofilm growth. After incubation, the plate was analysed by spectrophotometer using a VICTOR X5 multilabel plate reader set at the 600 nm (Perkin Elmer Wallac Victor 3 1420 Multilabe), to record OD600 values from the well suspensions before any washing or staining. These readings reflect total microbial growth (planktonic and loosely adherent cells), and were not used to quantify biofilm mass.

### Treatment with test solutions

**Figure 1:**
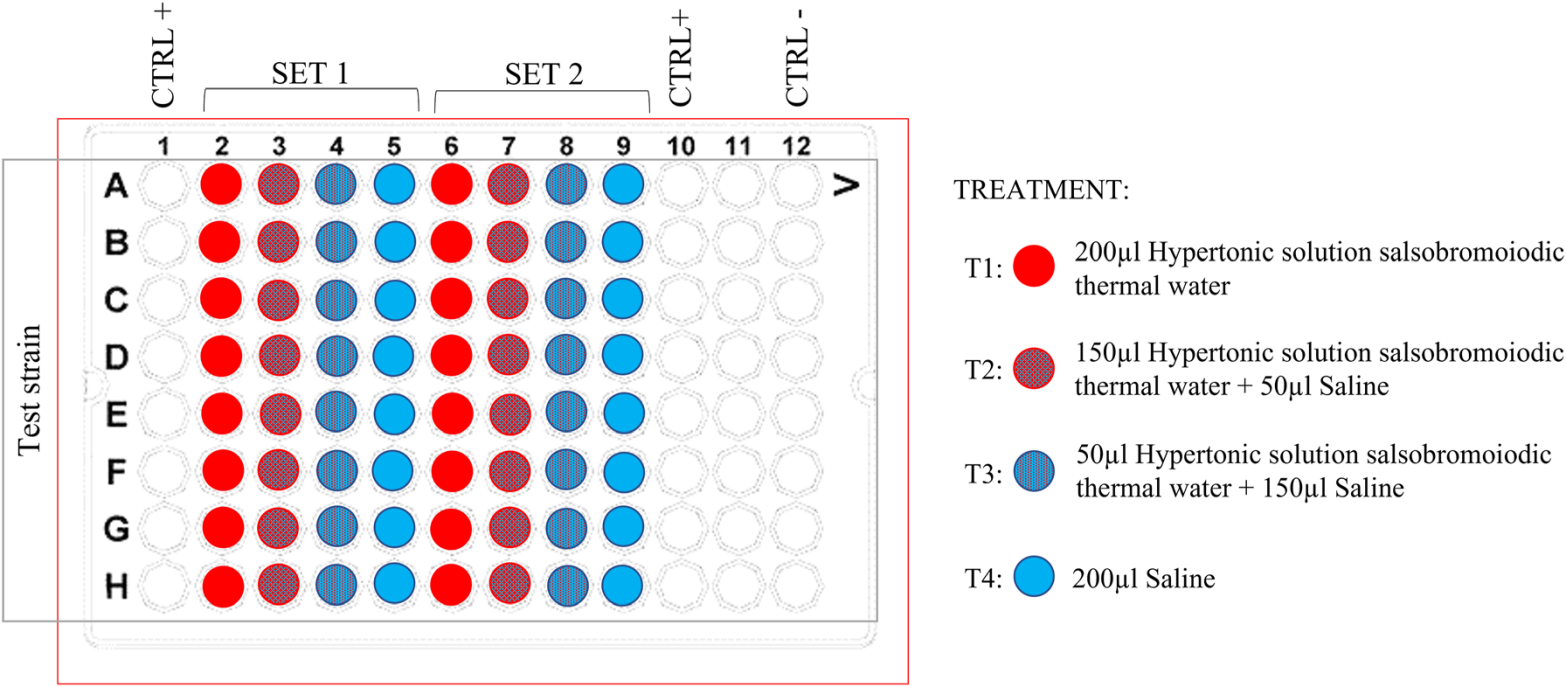
Experimental treatment scheme. Wells indicated as CTRL+ are wells in which the biofilm is not subjected to any treatment. CTRL− indicates wells without microbial inoculation (i.e., containing only culture medium and no cells), used as negative controls. Column 11 was intentionally left empty to prevent cross-contamination between positive and negative control wells located in adjacent columns.

Four different treatment conditions were applied to evaluate the dose-dependent effect of hypertonic salsobromoiodic thermal water on biofilm reduction: treatment 1 (T1) consisted of 200 µL of hypertonic salsobromoiodic thermal water, treatment 2 (T2) consisted of 150 µL of hypertonic salsobromoiodic thermal water combined with 50 µL of saline, treatment 3 (T3) consisted of 150 µL of saline combined with 50 µL of hypertonic salsobromoiodic thermal water, and treatment 4 (T4) consisted of 200 µL of saline (Figure 1).

Prior to treatment, both the wells and the biofilm-coated MBEC pegs were gently rinsed three times with 250 µL of sterile 0.9 % NaCl to remove non-adherent planktonic cells. Test solutions were dispensed into the wells of the MBEC plate according to the experimental scheme shown in Figure 1. Eight replicates were performed for each treatment, arranged as two sets of triplicates (SET 1 and SET 2) distributed in different sectors of the same plate (A–H).

To ensure controlled exposure and minimize interference from residual medium or planktonic biomass, a 96-well copy-plate was prepared following the same layout. The MBEC lid containing the biofilm-coated pegs was placed onto the copy-plate, and the pegs were immersed in the corresponding wells containing the test solutions. Plates were incubated at 37 °C under static conditions for 1 h.

### Crystal violet staining

Residual biofilm (i.e., the biofilm remaining after the treatment and washing steps) was quantified using the method proposed by Stepanović et al. with minor modifications [46]. Each well was emptied and washed three times with 250 µL of 0.9 % NaCl. The MBEC pegs were subjected to the same washing procedure by immersion in a prepared washing plate. Following washing, the MBEC plates and corresponding pegs were dried under the laminar flow of a biological fume hood in a tipping position for 1 h to fix the biofilm.

**Figure 2:**
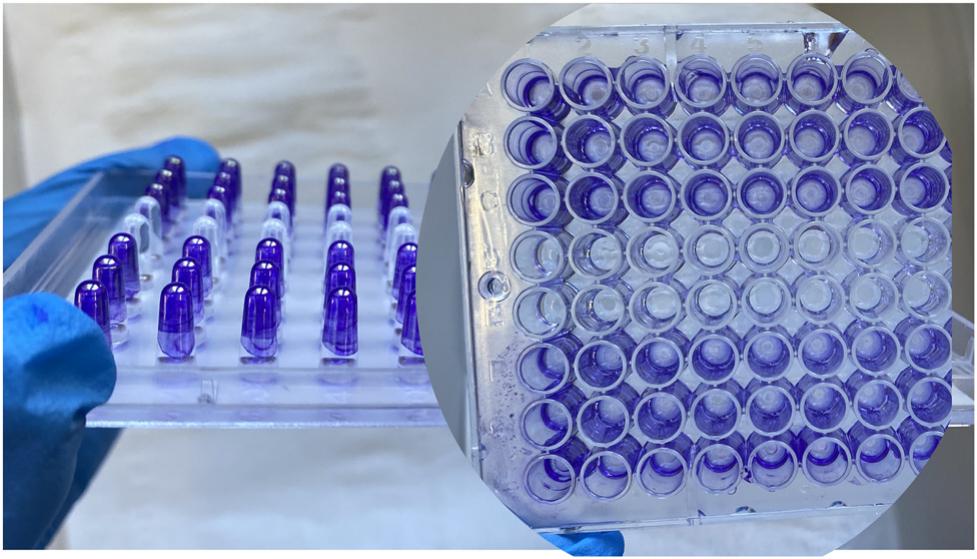
MBEC plate crystal violet staining (2 %). Pegs surface (left) and wells (right).

Subsequently, 250 µL of 2 % crystal violet solution was added to each well, the MBEC system was reassembled, and the plates were incubated for 5 min [47], 48]. Excess dye was removed by rinsing the plates under a moderate flow of tap water until the washing liquid was completely free of residual dye. Finally, to prevent unintended dilution of the crystal violet (2 %) during the subsequent solubilization step, the plates and pegs were dried again under laminar flow for 1 h (Figure 2).

### Biofilm breakdown from wells and pegs

The MBEC systems were filled with 250 µL of 0.9 % NaCl solution and subjected to two consecutive sonication steps (15 min each) at maximum intensity in an ultrasonic bath (Brandson) to evaluate biofilm detachment kinetics. After the first sonication, 50 µL of the resulting suspension from each well and from each peg was transferred into 200 µL of 33 % glacial acetic acid in separate 96-well plates to solubilize the crystal violet (2 %), following the protocol of Stepanović et al. [49]. This step allowed quantification of the easily detachable fraction of the residual biofilm.

Subsequently, the MBEC systems were restored to a final volume of 250 µL per well with 0.9 % NaCl, and a second sonication was performed under the same conditions to detach the more resistant biofilm still adhering to the wells and pegs. The resulting suspensions were again solubilized in 33 % glacial acetic acid and subjected to spectrophotometric measurement at 570 nm, the wavelength corresponding to the maximum absorbance peak of crystal violet, which allows accurate quantification of the dye bound to the biofilm matrix and, consequently, of the residual biofilm biomass. Measurements were performed using a multilabel plate (Perkin Elmer Wallac Victor 3 1420 Multilabel) [47], [48], [49]. This analysis enabled comparison between the early-detached and residual biofilm fractions. All measurements were performed in triplicate, and OD570 values were averaged across technical and biological replicates for both MBEC plates and pegs, providing a reliable estimation of the remaining biofilm biomass after treatment.

### Statistical analysis

The data collected in this study were analysed using statistical software (IBM SPSS Statistics v28.0). To assess the differences between the growth groups and their respective negative controls, analysis of variance (ANOVA) was applied with a significance level set at p<0.05. Unpaired Welch’s *t*-test further examined the statistical significance of the comparisons between the groups for normally distributed values, while the non-parametric Kolmogorov-Smirnov test was used for not-normally distributed values. The adequacy of the normal data distribution was assessed using the Shapiro-Wilk test with a significance level of α=0.05.

**Conflicts of Interest:** The authors declare no conflicts of interest.

**Ethical approval:** The conducted research is not related to either human or animals use.

## Results

### Evaluation of microbial growth in the MBEC system

**Figure 3:**
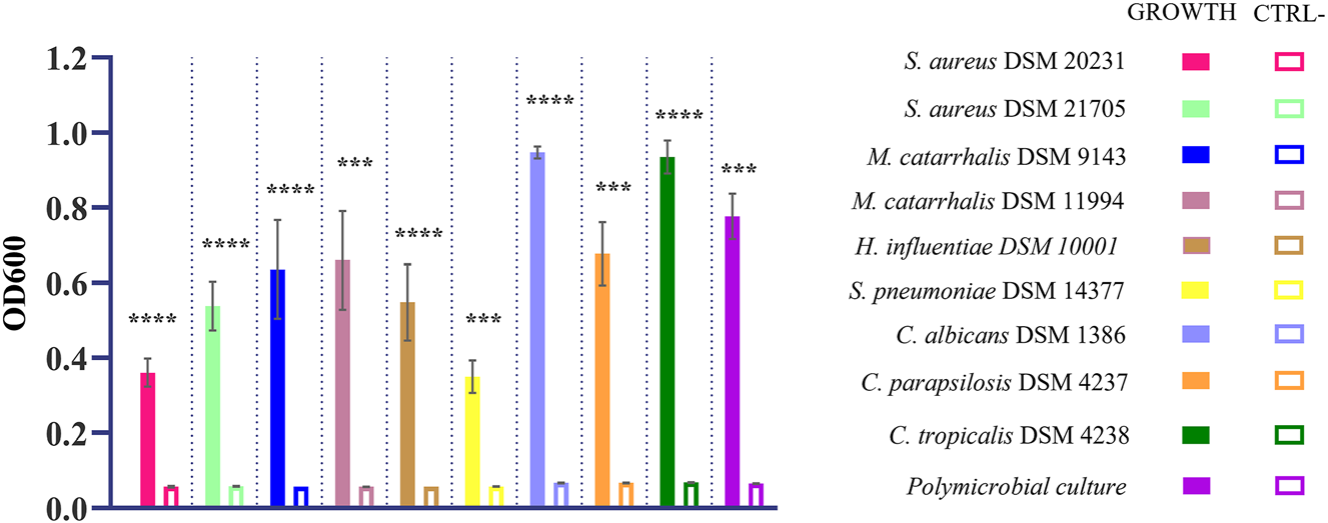
Growth of microorganisms in the MBEC system. The growth of each microorganism is reported in comparison with its own negative control (CTRL−) (***=p-value <0.01 or ****=p-value <0.001, Welch’s *t*-test and Kolmogorov-Smirnov test).

All bacterial and yeast strains and the polymicrobial culture exhibited statistically significant growth (OD600) in the MBEC system compared to the negative control (CTRL−), which consisted solely of the culture medium (Figure 3, Table 2).

**Table 2:** Evaluation of the microbial growth of each strain in the MBEC system.

Bacterial and yeast strains	OD600 mean (± D.S.)	OD600 CTRL−	p-Value
*S. aureus* DSM 20231	0.361 ± 0.037	0.057 ± 0.002	<0.0001
*S. aureus* DSM 21705	0.539 ± 0.065	0.058 ± 0.001	<0.0001
*M. catarrhalis* DSM 9143	0.636 ± 0.131	0.057 ± 0.001	<0.0001
*M. catarrhalis* DSM 11994	0.660 ± 0.131	0.057 ± 0.001	0.0002
*H. influenzae* DSM 10001	0.548 ± 0.102	0.057 ± 0.001	<0.0001
*S. pneumoniae* DSM 14377	0.350 ± 0.043	0.058 ± 0.001	0.0002
*C. albicans* DSM 1386	0.947 ± 0.016	0.067 ± 0.001	<0.0001
*C. parapsilosis* DSM 4237	0.677 ± 0.084	0.067 ± 0.001	0.0002
*C. tropicalis* DSM 4238	0.935 ± 0.044	0.068 ± 0.001	<0.0001
Polymicrobial culture	0.777 ± 0.060	0.066 ± 0.001	0.0002

Data are expressed as mean (± standard deviation) and compared to the culture medium’s negative control (CTRL−) alone. Statistical significance was determined using Welch’s parametric *t*-test for normally distributed data (p-value <0.0001) and the non-parametric Kolmogorov-Smirnov test for non-normally distributed data (p-value <0.001). OD600 readings were used as a semi-quantitative proxy for total microbial growth (including both planktonic and early adherent cells), with biofilm mass assessed separately through crystal violet staining at OD570.

Among the tested strains, *C. albicans* DSM 1386 demonstrated the highest mean growth (0.947 ± 0.016), followed by *C. tropicalis* DSM 4238 (0.935 ± 0.044). In contrast, *S. pneumoniae* DSM 14377 exhibited the lowest growth (0.350 ± 0.043). Polymicrobial cultures also showed substantial growth, with a mean value of 0.777 ± 0.060 (Figure 3, Table 2).

### Residual biofilm in wells and biofilm formation on MBEC pegs after treatment with test solutions

#### *S. aureus* DSM 20231

**Figure 4:**
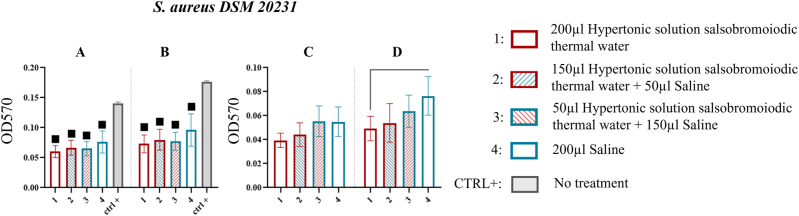
Comparison of biofilm reduction in *S. aureus* DSM 20231 across treatments and sonication times. In 4A and 4B, biofilm from the wells of the MBEC system after treatment and by 15 and 30 min of sonication have been reported. In 4C and 4D, the biofilm recovered from the pegs of the MBEC system after treatment and by 15 and 30 min of sonication. The numbers in the abscissa correspond to the treatments applied: 1=200 μL hypertonic solution salsobromoiodic thermal water, 2=150 μL hypertonic solution salsobromoiodic thermal water + 50 μL saline, 3=50 μL hypertonic solution salsobromoiodic thermal water + 150 μL saline, 4=200 μL saline. CTRL+ is the biofilm recovered from the wells undergoing no treatment. Squares indicate treatments that show a statistically significant difference compared to CTRL+, while lines connect treatments that differ significantly in terms of residual biofilm levels (ANOVA, p<0.05).

Analysis of MBEC system wells demonstrated that all treatments significantly reduced biofilm compared to untreated wells (CTRL+) (Figure 4A and B). After 15 min of sonication, the treatment with 200 µL of hypertonic solution salsobromoiodic thermal water (treatment 1) produced a biofilm residue of 0.060 ± 0.010 with a % reduction of 57.447, while the treatment with 200 µL of saline (treatment 4) showed a biofilm residue of 20.076 ± 0.018, compared to 0.141 ± 0.002 in the CTRL+ (Table 3).

**Table 3:** Residual biofilm biomass (OD570, mean ± SD) for *S. aureus* DSM 20231 after treatments and sonication.

Tested surface	Sonication time, min	Treatments				CTRL+
		Hypertonic solution salsobromoiodic thermal water (200 µL)	Hypertonic solution salsobromoiodic thermal water (150 µL) + saline (50 µL)	Hypertonic solution salsobromoiodic thermal water (50 µL) + saline (150 µL)	Saline (200 µL)	
Wells	15	0.060 ± 0.010	0.067 ± 0.012	0.065 ± 0.012	0.076 ± 0.018	0.141 ± 0.002
Wells	30	0.073 ± 0.015	0.080 ± 0.017	0.077 ± 0.015	0.096 ± 0.027	0.177 ± 0.002
Pegs	15	0.039 ± 0.006	0.044 ± 0.010	0.055 ± 0.013	0.055 ± 0.012	/
Pegs	30	0.049 ± 0.011	0.054 ± 0.016	0.064 ± 0.013	0.076 ± 0.016	/

The data, expressed as mean (± standard deviation), represent the residual biofilm recovered from the wells and pegs of the MBEC system after treatments with 15 and 30 min of sonication. CTRL+ indicates the biofilm recovered from the wells not subjected to treatment.

At 30 min, the biofilm residue for treatment 1 was 0.073 ± 0.015, lower than treatment 4 (0.096 ± 0.027) and the positive control (0.177 ± 0.002) (Table 3). The % reduction in treatment 4 vs. CTRL+ was 58.757. Although the differences between treatments were not statistically significant (p>0.05, ns). The data suggest that hypertonic solution salsobromoiodic thermal water (treatment 1) exhibited a greater capacity to disintegrate biofilm compared to saline (treatment 4), as evidenced by lower biofilm recovery (Figure 4A and B).

On the other hand, analysis of MBEC system pegs demonstrated a resembling behaviour (Figure 4C and D). After 15 min of sonication, a comparable biofilm amount was observed across all treatments. However, a slightly lower biofilm value was recorded for treatments 1 (0.039 ± 0.006) and 2 (0.044 ± 0.010). Following 30 min of sonication, treatment with hypertonic solution salsobromoiodic thermal water resulted in a biofilm residue of 0.049 ± 0.011, which was significantly lower compared to treatment 4 (0.076 ± 0.016, p<0.05) with a % reduction of 35.526 (Table 3).

#### *S. aureus* DSM 21705

**Figure 5:**
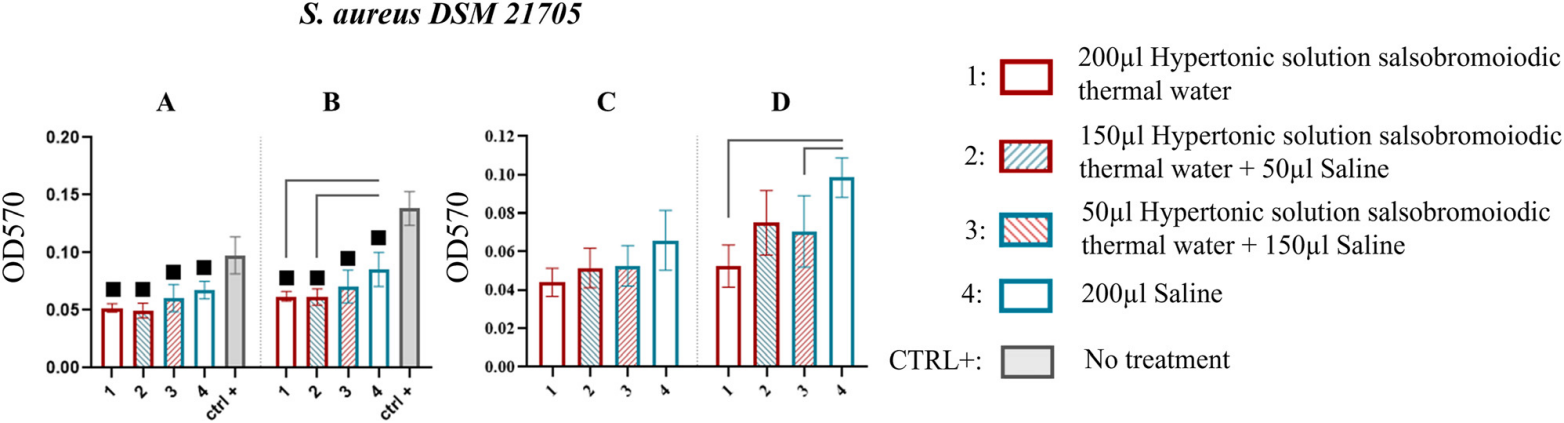
Comparison of biofilm reduction in *S. aureus* DSM 21705 across treatments. In 5A and 5B, biofilm from the wells of the MBEC system after treatment and by 15 and 30 min of sonication has been reported. In 5C and 5D, the biofilm recovered from the pegs of the MBEC system after treatment and by 15 and 30 min of sonication. The numbers in the abscissa correspond to the treatments applied: 1=200 μL hypertonic solution salsobromoiodic thermal water, 2=150 μL hypertonic solution salsobromoiodic thermal water + 50 μL saline, 3=50 μL hypertonic solution salsobromoiodic thermal water + 150 μL saline, 4=200 μL saline. CTRL+ is the biofilm recovered from the wells undergoing no treatment. Squares indicate treatments that show a statistically significant difference compared to CTRL+, while lines connect treatments that differ significantly in terms of residual biofilm levels (ANOVA, p<0.05).

The results from the MBEC system wells indicate that, for this strain, the biofilm recovered after all treatments was lower than that observed in the CTRL+ wells at both the first and second sonication steps (Figure 5A and B).

At the first sonication step, treatment 1 (0.052 ± 0.003) and treatment 2 (0.050 ± 0.006) show less residual biofilm than treatment 4 with saline alone (0.067 ± 0.008), although the differences are not statistically significant (p>0.05, ns) (Figure 5A, Table 4).

**Table 4:** Residual biofilm biomass (OD570, mean ± SD) for *S. aureus* DSM 21705 after treatments and sonication.

Tested surface	Sonication time, min	Treatments				CTRL+
		Hypertonic solution salsobromoiodic thermal water (200 µL)	Hypertonic solution salsobromoiodic thermal water (150 µL) + saline (50 µL)	Hypertonic solution salsobromoiodic thermal water (50 µL) + saline (150 µL)	Saline (200 µL)	
Wells	15	0.052 ± 0.003	0.050 ± 0.006	0.061 ± 0.012	0.067 ± 0.008	0.098 ± 0.016
Wells	30	0.062 ± 0.004	0.062 ± 0.007	0.071 ± 0.014	0.086 ± 0.015	0.138 ± 0.014
Pegs	15	0.044 ± 0.007	0.052 ± 0.010	0.053 ± 0.011	0.066 ± 0.015	/
Pegs	30	0.053 ± 0.011	0.075 ± 0.017	0.071 ± 0.018	0.099 ± 0.010	/

The data, expressed as mean (± standard deviation), represent the residual biofilm recovered from the wells and pegs of the MBEC system after treatments with 15 and 30 min of sonication. CTRL+ indicates the biofilm recovered from the wells not subjected to treatment.

This finding was further supported by statistical analysis at the second sonication step (p<0.05). Biofilm recovery after treatments 1 and 2 (0.062 ± 0.004 and 0.062 ± 0.007, respectively) is significantly lower than that obtained with treatment 4 (0.086 ± 0.015) with a % reduction of 27.907 (Figure 5B, Table 4).

The results obtained from the wells are further supported by the data collected from MBEC system pegs. At the first sonication step, no statistically significant differences are observed between treatments, but treatment 1 shows the lower biofilm recovery (0.044 ± 0.007) among treatments (Figure 5C, Table 4). Statistical analysis con-firms this trend at the second sonication step (p<0.05). Treatment 1 produced significantly less biofilm residue (0.053 ± 0.011) than treatment 4 (0.099 ± 0.010) with a % reduction of 46.464. Furthermore, treatment 3, primarily based on saline with the addition of a thermal component, demonstrated significantly lower biofilm recovery (0.071 ± 0.018) compared to saline alone (treatment 4) with a % reduction of 28.283 (Figure 5D, Table 4).

#### *M. catarrhalis* DSM 9143

**Figure 6:**
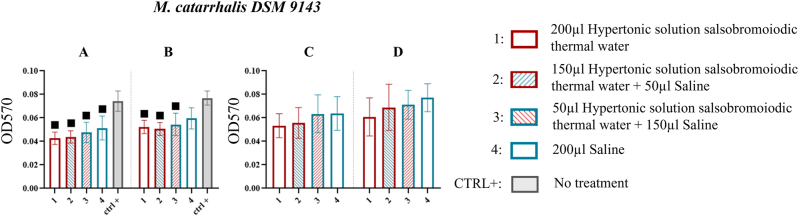
Comparison of biofilm reduction in *M. catarrhalis* DSM 9143 across treatments. In 6A and 6B biofilm from the wells of the MBEC system after treatment, and by 15 and 30 min of sonication has been reported. In 6C and 6D, the biofilm recovered from the pegs of the MBEC system after treatment and by 15 and 30 min of sonication. The numbers in the abscissa correspond to the treatments applied: 1=200 μL hypertonic solution salsobromoiodic thermal water, 2=150 μL hypertonic solution salsobromoiodic thermal water + 50 μL saline, 3=50 μL hypertonic solution salsobromoiodic thermal water + 150 μL saline, 4=200 μL saline. CTRL+ is the biofilm recovered from the wells undergoing no treatment. Squares indicate treatments that show a statistically significant difference compared to CTRL+, while lines connect treatments that differ significantly in terms of residual biofilm levels (ANOVA, p<0.05).

In the MBEC system wells, while no statistically significant differences were observed among the various treatments, a greater tendency toward biofilm reduction was noted with the solution containing hypertonic solution salsobromoiodic thermal water (0.043 ± 0.005) with a % reduction of 42.667 compared to the saline solution alone (0.052 ± 0.010) with a % reduction of 30.667, at the first sonication step (Figure 6A, Table 5).

**Table 5:** Residual biofilm biomass (OD570, mean ± SD) for *M. catarrhalis* DSM 9143 after treatments and sonication.

Tested surface	Sonication time, min	Treatments				CTRL+
		Hypertonic solution salsobromoiodic thermal water (200 µL)	Hypertonic solution salsobromoiodic thermal water (150 µL) + saline (50 µL)	Hypertonic solution salsobromoiodic thermal water (50 µL) + saline (150 µL)	Saline (200 µL)	
Wells	15	0.043 ± 0.005	0.044 ± 0.005	0.048 ± 0.009	0.052 ± 0.010	0.075 ± 0.009
Wells	30	0.052 ± 0.006	0.051 ± 0.006	0.055 ± 0.009	0.060 ± 0.009	0.077 ± 0.006
Pegs	15	0.053 ± 0.010	0.056 ± 0.013	0.064 ± 0.016	0.064 ± 0.014	/
Pegs	30	0.061 ± 0.016	0.069 ± 0.019	0.071 ± 0.012	0.077 ± 0.012	/

The data, expressed as mean (± standard deviation), represent the residual biofilm recovered from the wells and pegs of the MBEC system after treatments with 15 and 30 min of sonication. CTRL+ indicates the biofilm recovered from the wells not subjected to treatment.

This trend was further confirmed at the second sonication step, where only the reduction achieved by treatments containing hypertonic solution salsobromoiodic thermal water was significant (0.052 ± 0.006) when compared to the CTRL+ (0.077 ± 0.006; p<0.05) with a % reduction of 32.467 (Figure 6B, Table 5).

Similar biofilm recovery values were observed across treatments at both sonication steps for the pegs. However, a greater reduction in biofilm, although not statistically significant (p>0.05, ns), was recorded with the more concentrated hypertonic solution salsobromoiodic thermal water treatments (treatment 1: 0.053 ± 0.010; treatment 2: 0.056 ± 0.013) compared to the saline-only treatment (treatment 4: 0.064 ± 0.016) at the first sonication time (Figure 6C, Table 5). At the second sonication step, a similar trend was evident. Treatment 1 yielded a biofilm residue of 0.061 ± 0.016, followed by treatment 2 (0.069 ± 0.019), with both showing lower values than treatment 4 (0.077 ± 0.012) (Figure 6D, Table 5).

#### *M. catarrhalis* DSM 11994

**Figure 7:**
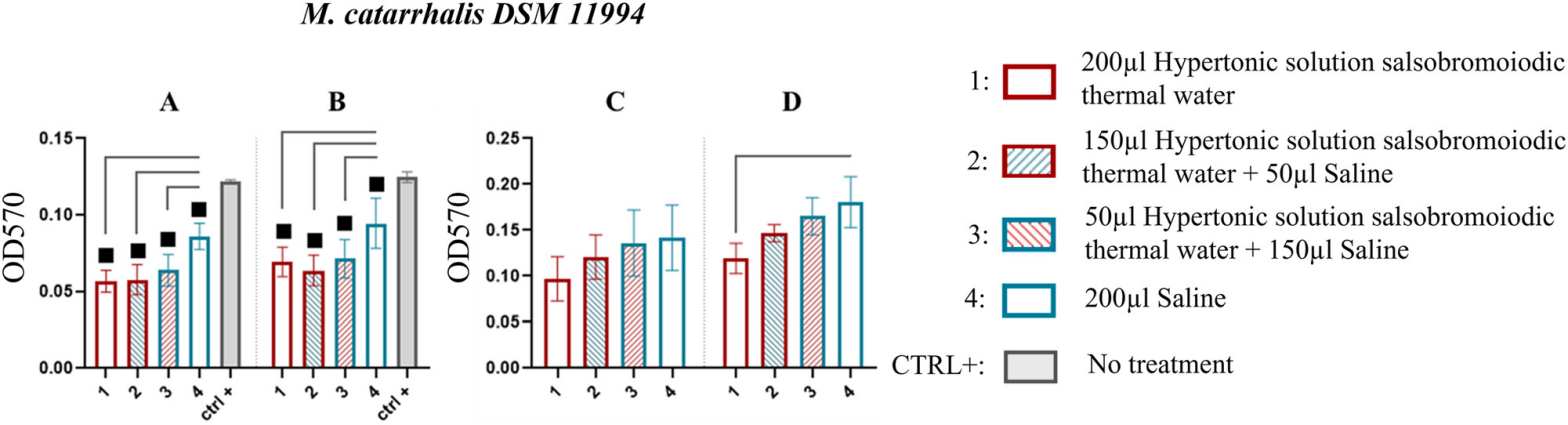
Comparison of biofilm reduction in *M. catarrhalis* DSM 11994 across treatments. In 7A and 7B, the biofilm from the wells of the MBEC system after treatment, and by 15 and 30 min of sonication has been reported. In 7C and 7D, the biofilm recovered from the pegs of the MBEC system after treatment and by 15 and 30 min of sonication. The numbers in the abscissa correspond to the treatments applied: 1=200 μL hypertonic solution salsobromoiodic thermal water, 2=150 μL hypertonic solution salsobromoiodic thermal water + 50 μL saline, 3=50 μL hypertonic solution salsobromoiodic thermal water + 150 μL saline, 4=200 μL saline. CTRL+ is the biofilm recovered from the wells undergoing no treatment. Squares indicate treatments that show a statistically significant difference compared to CTRL+, while lines connect treatments that differ significantly in terms of residual biofilm levels (ANOVA, p<0.05).

MBEC system wells for *M. catarrhalis* DSM 11994 revealed that treatments 1, 2, and 3 significantly reduced biofilm compared to CTRL+ (p<0.05) (Figure 7A and B). After 15 min (first sonication), the residual biofilm was 0.057 ± 0.007 (treatment 1) with a % reduction of 53.279, 0.058 ± 0.010 (treatment 2) with a % reduction of 52.459, and 0.064 ± 0.010 (treatment 3) with a % reduction of 47.541, compared to 0.122 ± 0.001 in the CTRL+. Following 30 min (second sonication), the residual biofilm was 0.069 ± 0.010 (treatment 1) with a % reduction of 44.800, 0.064 ± 0.010 (treatment 2) with a % reduction of 48.800, and 0.071 ± 0.013 (treatment 3) with a % reduction of 43.200, compared to CTRL+ (Table 6).

**Table 6:** Residual biofilm biomass (OD570, mean ± SD) for *M. catarrhalis* DSM 11994 after treatments and sonication.

Tested surface	Sonication time, min	Treatments				CTRL+
		Hypertonic solution salsobromoiodic thermal water (200 µL)	Hypertonic solution salsobromoiodic thermal water (150 µL) + saline (50 µL)	Hypertonic solution salsobromoiodic thermal water (50 µL) + saline (150 µL)	Saline (200 µL)	
Wells	15	0.057 ± 0.007	0.058 ± 0.010	0.064 ± 0.010	0.086 ± 0.008	0.122 ± 0.001
Wells	30	0.069 ± 0.010	0.064 ± 0.010	0.071 ± 0.013	0.095 ± 0.016	0.125 ± 0.003
Pegs	15	0.097 ± 0.024	0.121 ± 0.024	0.136 ± 0.036	0.142 ± 0.036	/
Pegs	30	0.119 ± 0.016	0.147 ± 0.094	0.165 ± 0.020	0.181 ± 0.028	/

The data, expressed as mean (± standard deviation), represent the residual biofilm recovered from the wells and pegs of the MBEC system after treatments with 15 and 30 min of sonication. CTRL+ indicates the biofilm recovered from the wells not subjected to treatment.

Regarding the pegs, no significant differences were observed in the biofilm recovery between treatments at the first sonication step. However, a clear trend of all hypertonic solution salsobromoiodic thermal water (treatments 1, 2, and 3) reducing biofilm in a manner proportional to the percentage of thermal water was noted (Figure 7C, Table 6). At the second sonication step, the anti-biofilm effect of hypertonic solution salsobromoiodic thermal water was more pronounced and statistically confirmed (p<0.05). Treatment 1 resulted in significantly less biofilm recovery (0.119 ± 0.016) compared to treatment 4 (0.181 ± 0.028) with a % reduction of 34.254 (Figure 7D, Table 6).

#### *H. influenzae* DSM 10001

**Figure 8:**
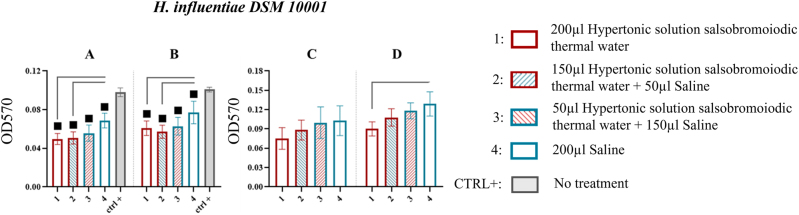
Comparison of biofilm reduction in *H. influenzae* DSM 10001 across treatments. In 8A and 8B biofilm from the wells of the MBEC system after treatment and by 15 and 30 min of sonication has been reported. In 8C and 8D, the biofilm recovered from the pegs after treatment and by 15 and 30 min of sonication. The numbers in the abscissa correspond to the treatments applied: 1=200 μL hypertonic solution salsobromoiodic thermal water, 2=150 μL hypertonic solution salsobromoiodic thermal water + 50 μL saline, 3=50 μL hypertonic solution salsobromoiodic thermal water + 150 μL saline, 4=200 μL saline. CTRL+ is the biofilm recovered from the wells undergoing no treatment. Squares indicate treatments that show a statistically significant difference compared to CTRL+, while lines connect treatments that differ significantly in terms of residual biofilm levels (ANOVA, p<0.05).

MBEC system wells revealed that all treatments, at the first and second sonication steps, resulted in significantly lower biofilm recovery than the CTRL+ (p<0.05). During the first sonication step, biofilm recovery values in the wells were 0.050 ± 0.006 (treatment 1) with a % reduction of 28.571 and 0.051 ± 0.006 (treatment 2) with a % reduction of 27.143, compared to 0.070 ± 0.007 for treatment 4 (p<0.05). In the second sonication step, the residual biofilm was 0.061 ± 0.007 (treatment 1) with a % reduction of 20.780 and 0.057 ± 0.007 (treatment 2) with a % reduction of 25.974, compared to 0.077 ± 0.012 for treatment 4 (p<0.05) (Figure 8A and B, Table 7).

**Table 7:** Residual biofilm biomass (OD570, mean ± SD) for *H. influenzae* DSM 10001 after treatments and sonication.

Tested surface	Sonication time, min	Treatments				CTRL+
		Hypertonic solution salsobromoiodic thermal water (200 µL)	Hypertonic solution salsobromoiodic thermal water (150 µL) + saline (50 µL)	Hypertonic solution salsobromoiodic thermal water (50 µL) + saline (150 µL)	Saline (200 µL)	
Wells	15	0.050 ± 0.006	0.051 ± 0.006	0.056 ± 0.008	0.070 ± 0.007	0.098 ± 0.004
Wells	30	0.061 ± 0.007	0.057 ± 0.007	0.063 ± 0.091	0.077 ± 0.012	0.101 ± 0.002
Pegs	15	0.075 ± 0.017	0.088 ± 0.016	0.100 ± 0.025	0.103 ± 0.023	/
Pegs	30	0.090 ± 0.011	0.108 ± 0.013	0.118 ± 0.012	0.129 ± 0.019	/

The data, expressed as mean (± standard deviation), represent the residual biofilm recovered from the wells and pegs of the MBEC system after treatments with 15 and 30 min of sonication. CTRL+ indicates the biofilm recovered from the wells not subjected to treatment.

For the pegs, a trend of biofilm reduction proportional to the concentration of hypertonic solution salsobromoiodic thermal water was observed. This was confirmed by a significant difference between treatment 1 (0.090 ± 0.011) and treatment 4 (0.129 ± 0.019) with a % reduction of 30.232 (Figure 8C and D, Table 7).

#### *S. pneumoniae* DSM 14377

**Figure 9:**
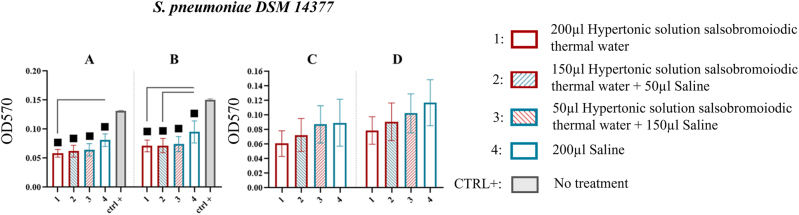
Comparison of biofilm reduction in *S. pneumoniae* DSM 14377 across treatments. In 9A and 9B biofilm detached from the wells of the MBEC system after treatment, and by 15 and 30 min of sonication has been reported. In 9C and 9D the biofilm recovered from the pegs after treatment, and by 15 and 30 min of sonication, respectively. The numbers in the abscissa correspond to the treatments applied: 1=200 μL hypertonic solution salsobromoiodic thermal water, 2=150 μL hypertonic solution salsobromoiodic thermal water + 50 μL saline, 3=50 μL hypertonic solution salsobromoiodic thermal water + 150 μL saline, 4=200 μL saline. CTRL+ is the biofilm recovered from the wells undergoing no treatment. Squares indicate treatments that show a statistically significant difference compared to CTRL+, while lines connect treatments that differ significantly in terms of residual biofilm levels (ANOVA, p<0.05).

For *S. pneumoniae* DSM 14377, results from the MBEC system wells showed that all treatments significantly reduced biofilm compared to CTRL+ (p<0.05). At the first sonication step, residual biofilm was significantly lower for treatment 1 (0.058 ± 0.007) with a % reduction of 28.395 compared to treatment 4 (0.081 ± 0.011) (Figure 9A, Table 8).

**Table 8:** Residual biofilm biomass (OD570, mean ± SD) for *S. pneumoniae* DSM 14377 after treatments and sonication.

Tested surface	Sonication time, min	Treatments				CTRL+
		Hypertonic solution salsobromoiodic thermal water (200 µL)	Hypertonic solution salsobromoiodic thermal water (150 µL) + saline (50 µL)	Hypertonic solution salsobromoiodic thermal water (50 µL) + saline (150 µL)	Saline (200 µL)	
Wells	15	0.058 ± 0.007	0.062 ± 0.010	0.065 ± 0.011	0.081 ± 0.011	0.131 ± 0.001
Wells	30	0.071 ± 0.010	0.072 ± 0.012	0.074 ± 0.013	0.095 ± 0.019	0.151 ± 0.002
Pegs	15	0.061 ± 0.018	0.073 ± 0.023	0.087 ± 0.026	0.089 ± 0.032	/
Pegs	30	0.079 ± 0.019	0.091 ± 0.026	0.102 ± 0.027	0.117 ± 0.032	/

The data, expressed as mean (± standard deviation), represent the residual biofilm recovered from the wells and pegs of the MBEC system after treatments with 15 and 30 min of sonication. CTRL+ indicates the biofilm recovered from the wells not subjected to treatment.

Similarly, during the second sonication step, biofilm residue was significantly lower for treatment 1 (0.071 ± 0.010) with a % reduction of 25.263 compared to treatment 4 (0.095 ± 0.019), as well as for treatment 2 (0.072 ± 0.012) with a % reduction of 24.210 compared to treatment 4 (0.095 ± 0.019) (Figure 9B, Table 8).

In the pegs, while biofilm reduction proportional to the concentration of hypertonic solution salsobromoiodic thermal water was observed, no statistically significant differences were recorded (Figure 9C and D, Table 8).

#### *C. albicans* DSM 1386

**Figure 10:**
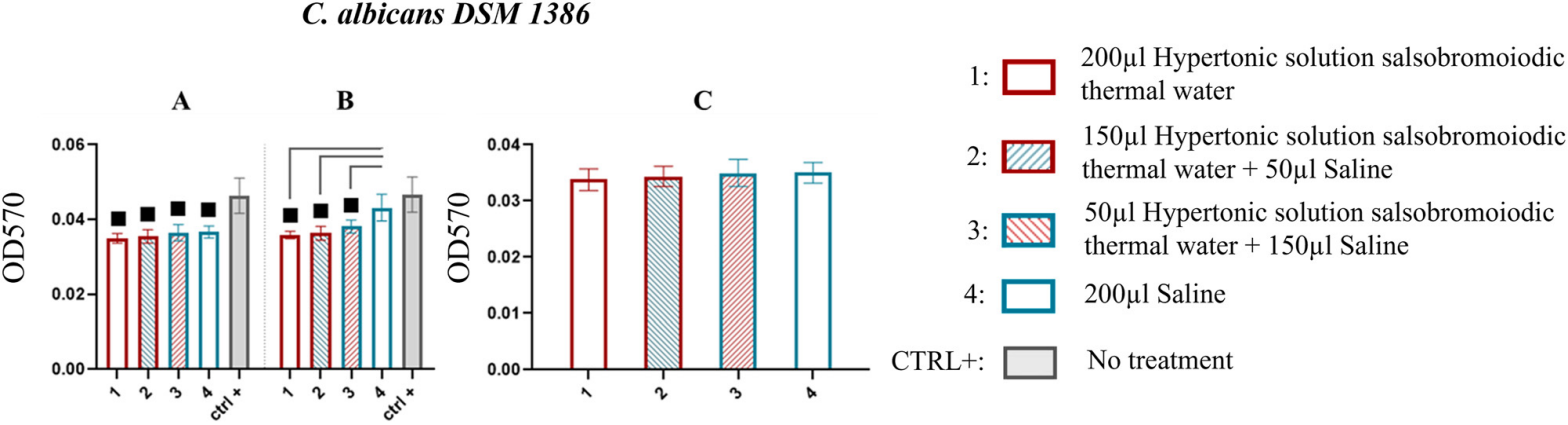
Comparison of biofilm reduction in *C. albicans* DSM 1386 across treatments. In 10A and 10B, the biofilm detached from the wells of the MBEC system after treatment and by 15 and 30 min of sonication has been reported. In 10C and 10D, the biofilm recovered from the pegs after treatment and by 15 min of sonication. The numbers in the abscissa correspond to the treatments applied: 1=200 μL hypertonic solution salsobromoiodic thermal water, 2=150 μL hypertonic solution salsobromoiodic thermal water + 50 μL saline, 3=50 μL hypertonic solution salsobromoiodic thermal water + 150 μL saline, 4=200 μL saline. CTRL+ is the biofilm recovered from the wells undergoing no treatment. Squares indicate treatments that show a statistically significant difference compared to CTRL+, while lines connect treatments that differ significantly in terms of residual biofilm levels (ANOVA, p<0.05).

At the first sonication step (15 min), results from the MBEC system wells showed that all treatments (treatment 1: 0.035 ± 0.001, treatment 2: 0.036 ± 0.002, treatment 3: 0.037 ± 0.002, treatment 4: 0.037 ± 0.002) resulted in significantly lower biofilm recovery compared to the control (CTRL+: 0.046 ± 0.005; p<0.05) (Figure 10A, Table 9).

**Table 9:** Residual biofilm biomass (OD570, mean ± SD) for *C. albicans* DSM 1386 after treatments and sonication.

Tested surface	Sonication time, min	Treatments				CTRL+
		Hypertonic solution salsobromoiodic thermal water (200 µL)	Hypertonic solution salsobromoiodic thermal water (150 µL) + saline (50 µL)	Hypertonic solution salsobromoiodic thermal water (50 µL) + saline (150 µL)	Saline (200 µL)	
Wells	15	0.035 ± 0.001	0.036 ± 0.002	0.037 ± 0.002	0.037 ± 0.002	0.046 ± 0.005
Wells	30	0.036 ± 0.001	0.036 ± 0.002	0.038 ± 0.002	0.043 ± 0.005	0.047 ± 0.005
Pegs	15	0.034 ± 0.002	0.034 ± 0.002	0.035 ± 0.002	0.035 ± 0.002	/
Pegs	30	/	/	/	/	/

The data, expressed as mean (± standard deviation), represent the residual biofilm recovered from the wells and pegs of the MBEC system after treatments with 15 and 30 min of sonication. CTRL+ indicates the biofilm recovered from the wells not subjected to treatment.

The % reduction observed was 23.913 for treatment 1, 21.739 for treatment 2, 19.565 for treatment 3 and 19.565 for treatment 4 compared to CTRL+. At the second sonication step (30 min), all solutions containing hypertonic solution salsobromoiodic thermal water showed significantly lower biofilm recovery compared to both the un-treated control and the saline solution (p<0.05). The biofilm recovery values were 0.036 ± 0.001 (treatment 1) with a % reduction of 23.404, 0.036 ± 0.002 (treatment 2) with a % reduction of 23.404, and 0.038 ± 0.002 (treatment 3) with a % reduction of 19.149, compared to 0.047 ± 0.005 (CTRL+) (Figure 10B, Table 9). In contrast, saline alone showed recovery levels comparable to the untreated control.

For the pegs, biofilm recovery was evaluated only at the first sonication step, as no residual biofilm was detected after this step. At 15 min, no significant differences were found among treatments (p>0.05, ns) (Figure 10C, Table 9).

#### *C. parapsilosis* DSM 4237

**Figure 11:**
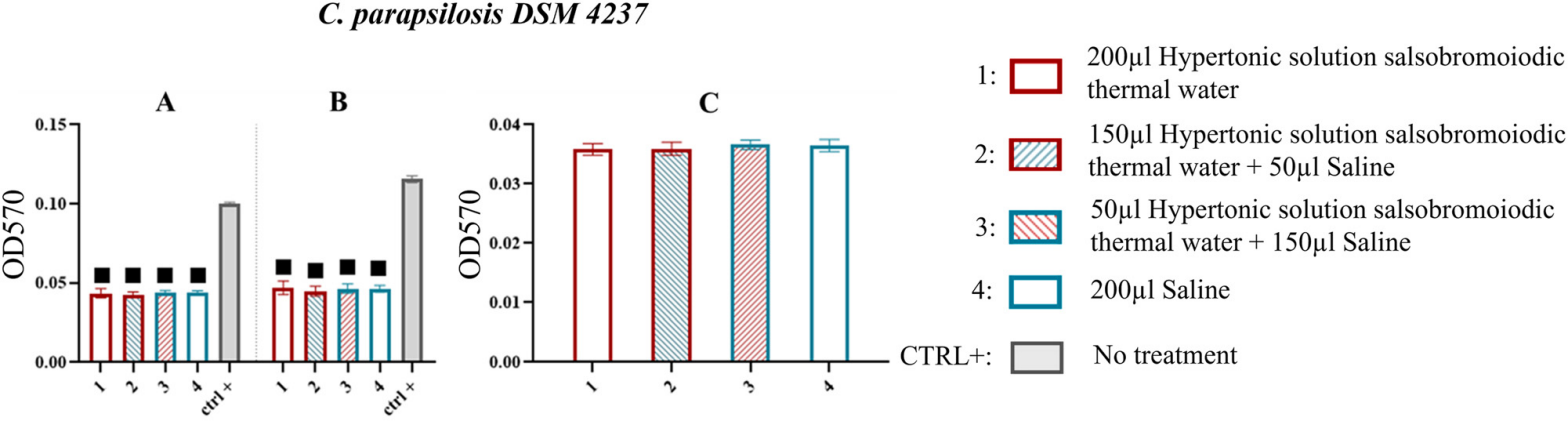
Comparison of biofilm reduction in *C. parapsilosis* DSM 4237 across treatments. In 11A and 11B, biofilm from the wells of the MBEC system after treatment and by 15 and 30 min of sonication has been reported. In 13C and 13D the biofilm recovered from the pegs of the MBEC system after treatment and 15 min of sonication. The numbers in the abscissa correspond to the treatments applied: 1=200 μL hypertonic solution salsobromoiodic thermal water, 2=150 μL hypertonic solution salsobromoiodic thermal water + 50 μL saline, 3=50 μL hypertonic solution salsobromoiodic thermal water + 150 μL saline, 4=200 μL saline. CTRL+ is the biofilm recovered from the wells undergoing no treatment. Squares indicate treatments that show a statistically significant difference compared to CTRL+, while lines connect treatments that differ significantly in terms of residual biofilm levels (ANOVA, p<0.05).

For *C. parapsilosis* DSM 4237, all treatments applied in the MBEC system wells significantly reduced biofilm compared to the CTRL+. In particular, at the first sonification step a % reduction of 56.000 was observed in treatment 1 compared to CTRL+ and at the second step a % reduction of 59.483 in treatment 1 compared to CTRL+. However, no significant differences were observed among treatments at both sonication steps (Figure 11A and B, Table 10).

**Table 10:** Residual biofilm biomass (OD570, mean ± SD) for *C. parapsilosis* DSM 4237 after treatments and sonication.

Tested surface	Sonication time, min	Treatments				CTRL+
		Hypertonic solution salsobromoiodic thermal water (200 µL)	Hypertonic solution salsobromoiodic thermal water (150 µL) + saline (50 µL)	Hypertonic solution salsobromoiodic thermal water (50 µL) + saline (150 µL)	Saline (200 µL)	
Wells	15	0.044 ± 0.003	0.043 ± 0.002	0.044 ± 0.002	0.044 ± 0.002	0.100 ± 0.026
Wells	30	0.047 ± 0.004	0.045 ± 0.003	0.046 ± 0.003	0.046 ± 0.002	0.116 ± 0.037
Pegs	15	0.036 ± 0.001	0.036 ± 0.001	0.037 ± 0.001	0.036 ± 0.001	/
Pegs	30	/	/	/	/	/

The data, expressed as mean (± standard deviation), represent the residual biofilm recovered from the wells and pegs of the MBEC system after treatments with 15 and 30 min of sonication. CTRL+ indicates the biofilm recovered from the wells not subjected to treatment.

Similarly, in the peg system, evaluated only at the first sonication step, no significant differences were noted among treatments (Figure 11C, Table 10).

#### *C. tropicalis* DSM 4238

**Figure 12:**
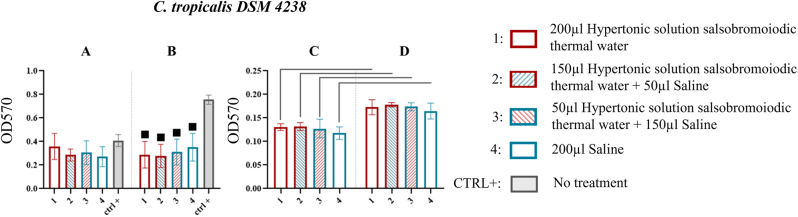
Comparison of biofilm reduction in *C. tropicalis* DSM 4238 across treatments. In 12A and 12B, biofilm detached from the wells of the MBEC system after treatment and by 15 and 30 min of sonication has been reported. In 12C and 12D the biofilm recovered from the pegs of the MBEC system after treatment and by 15 and 30 min of sonication respectively. The numbers in the abscissa correspond to the treatments applied: 1=200 μL hypertonic solution salsobromoiodic thermal water, 2=150 μL hypertonic solution salsobromoiodic thermal water + 50 μL saline, 3=50 μL hypertonic solution salsobromoiodic thermal water + 150 μL saline, 4=200 μL saline. CTRL+ is the biofilm recovered from the wells undergoing no treatment. Squares indicate treatments that show a statistically significant difference compared to CTRL+, while lines connect treatments that differ significantly in terms of residual biofilm levels (ANOVA, p<0.05).

Data from the MBEC system wells for *C. tropicalis* DSM 4238 showed that at the first sonication step (15 min), biofilm recovery was not significantly influenced by the treatments, with values similar to the CTRL+ (Figure 12A, Table 11).

**Table 11:** Residual biofilm biomass (OD570, mean ± SD) for *C. tropicalis* DSM 4238 after treatments and sonication.

Tested surface	Sonication time, min	Treatments				CTRL+
		Hypertonic solution salsobromoiodic thermal water (200 µL)	Hypertonic solution salsobromoiodic thermal water (150 µL) + saline (50 µL)	Hypertonic solution salsobromoiodic thermal water (50 µL) + saline (150 µL)	Saline (200 µL)	
Wells	15	0.358 ± 0.111	0.285 ± 0.052	0.305 ± 0.101	0.271 ± 0.085	0.409 ± 0.051
Wells	30	0.288 ± 0.117	0.278 ± 0.099	0.311 ± 0.109	0.352 ± 0.161	0.756 ± 0.230
Pegs	15	0.131 ± 0.028	0.132 ± 0.024	0.128 ± 0.020	0.118 ± 0.042	/
Pegs	30	0.173 ± 0.038	0.178 ± 0.033	0.174 ± 0.032	0.165 ± 0.025	/

The data, expressed as mean (± standard deviation), represent the residual biofilm recovered from the wells and pegs of the MBEC system after treatments with 15 and 30 min of sonication. CTRL+ indicates the biofilm recovered from the wells not subjected to treatment.

However, at the second sonication step (30 min), treatments significantly reduced biofilm recovery compared to the CTRL+: 0.756 ± 0.230, with values of 0.288 ± 0.117 (treatment 1) with a % reduction of 61.905, 0.278 ± 0.099 (treatment 2) with a % reduction of 63.227, 0.311 ± 0.109 (treatment 3) with a % reduction of 58.862, and 0.352 ± 0.161 (treatment 4) with a % reduction of 53.439 (Figure 12B, Table 11).

No significant differences were observed between treatments at either sonication step for the pegs. However, a significant increase in biofilm recovery was recorded as a function of sonication time for each treatment (e.g., treatment 1: 0.131 ± 0.028 at 15 min vs. 0.173 ± 0.038 at 30 min) (Figure 12C and D, Table 11).

#### Polymicrobial culture

**Figure 13:**
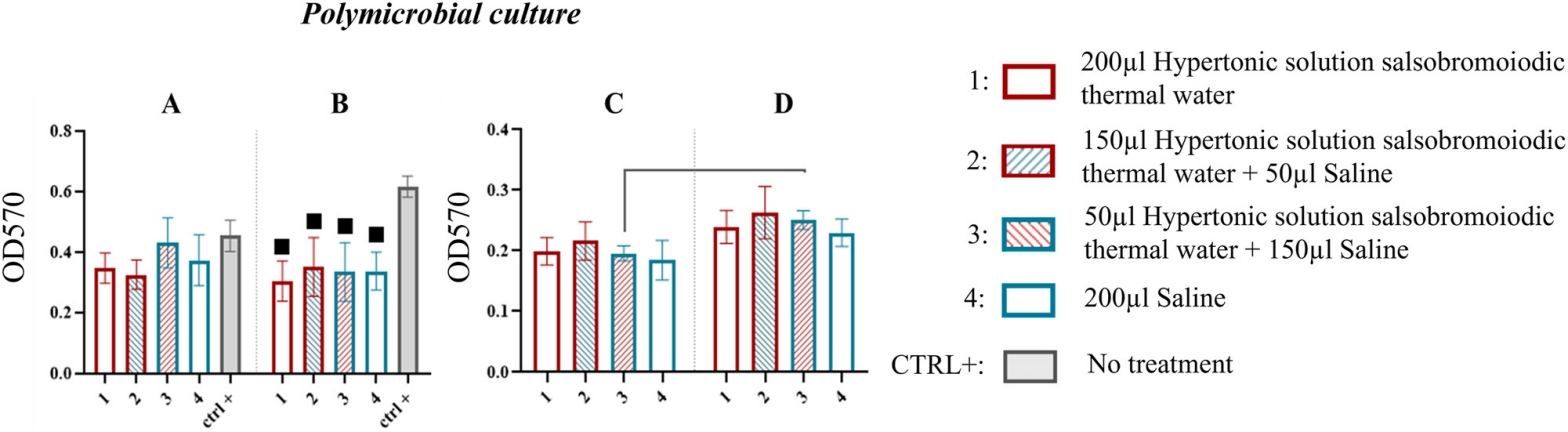
Comparison of biofilm reduction in polymicrobial culture across treatments. In 13A and 13B, biofilm from the MBEC system pegs after treatment and by 15 and 30 min of sonication has been reported. In 13C and 13D the biofilm recovered from the pegs of the MBEC system after treatment and by 15 and 30 min of sonication respectively. The numbers in the abscissa correspond to the treatments applied: 1=200 μL hypertonic solution salsobromoiodic thermal water, 2=150 μL hypertonic solution salsobromoiodic thermal water + 50 μL saline, 3=50 μL hypertonic solution salsobromoiodic thermal water + 150 μL saline, 4=200 μL saline. CTRL+ is the biofilm re-covered from the wells undergoing no treatment. Squares indicate treatments that show a statistically significant difference compared to CTRL+, while lines connect treatments that differ significantly in terms of residual biofilm levels (ANOVA, p<0.05).

At the first sonication step (15 min), the MBEC system wells showed that no treatment significantly affected biofilm recovery compared to the CTRL+ (p>0.05, ns) (Figure 13A, Table 12).

**Table 12:** Residual biofilm biomass (OD570, mean ± SD) for polymicrobial culture after treatments and sonication.

Tested surface	Sonication time, min	Treatments				CTRL+
		Hypertonic solution salsobromoiodic thermal water (200 µL)	Hypertonic solution salsobromoiodic thermal water (150 µL) + saline (50 µL)	Hypertonic solution salsobromoiodic thermal water (50 µL) + saline (150 µL)	Saline (200 µL)	
Wells	15	0.347 ± 0.050	0.325 ± 0.048	0.430 ± 0.243	0.373 ± 0.098	0.454 ± 0.052
Wells	30	0.304 ± 0.077	0.351 ± 0.114	0.334 ± 0.225	0.337 ± 0.072	0.616 ± 0.026
Pegs	15	0.199 ± 0.063	0.216 ± 0.075	0.196 ± 0.033	0.184 ± 0.066	/
Pegs	30	0.239 ± 0.086	0.263 ± 0.108	0.251 ± 0.046	0.230 ± 0.113	/

The data, expressed as mean (± standard deviation), represent the residual biofilm recovered from the wells and pegs of the MBEC system after treatments with 15 and 30 min of sonication. CTRL+ indicates the biofilm recovered from the wells not subjected to treatment.

Conversely, at the second sonication step (30 min), all treatments significantly reduced biofilm recovery compared to the CTRL+: 0.616 ± 0.026. Biofilm recovery values were 0.304 ± 0.077 (treatment 1) with a % reduction of 50.649, 0.351 ± 0.114 (treatment 2) with a % reduction of 43.019, 0.334 ± 0.225 (treatment 3) with a % reduction of 45.780, and 0.337 ± 0.072 (treatment 4) with a % reduction of 45.292 compared to the CTRL+, although no significant differences were found among treatments (Figure 13B, Table 12).

For the pegs, comparable biofilm recovery values were observed across treatments at both sonication steps. However, at the second sonication step (30 min), treatment 3 showed a significant increase in biofilm recovery (15 min: 0.196 ± 0.033 vs. 30 min: 0.251 ± 0.046) with a % reduction of 21.912. Overall, no significant differences were found in the pegs (Figure 13C and D, Table 12).

## Discussion

In the present study, we found that the physicochemical properties of hypertonic salsobromoiodic thermal water confer a concentration-dependent antibiofilm effect across different microbial communities. This result provides a coherent framework for understanding the observed differences in biofilm resilience, experimental outcomes, and microbial susceptibility. Biofilms are structured microbial communities encased in a self-produced extra-cellular matrix that adheres to mucosal surfaces such as the nasal cavity [4], 50]. Their formation is significantly associated with chronic nasal infections, contributing to persistent inflammation and resistance to conventional therapies [51], 52]. According to the National Institutes of Health (NIH), 65–80 % of infections treated in developed countries may be attributable to microorganisms growing within biofilms [53]. In children with recurrent upper respiratory tract infections, patients with nasal polyposis or septal deviations biofilms can colonize up to 95 % of the nasopharynx [54], [55], [56]. The resistance of biofilms to antibiotic therapy is primarily due to mechanisms such us limited antibiotic diffusion, through the extracellular matrix; the presence of dormant microbial cells in nutrient and oxygen-depleted zone; and enhanced horizontal gene transfer [27], [57], [58], [59], [60], [61]. These strategies can render biofilm-associated bacteria up to 1,000 times more resistant to antibiotics compared to their planktonic counterparts [27], 28].

Conventional antimicrobial tests, including the minimum inhibitory concentration (MIC) and minimum bactericidal concentration (MBC), are limited in their ability to evaluate biofilm-associated microorganisms, as they primarily measure the susceptibility of planktonic cells. Microbes embedded in biofilms are encased within an extracellular polymeric matrix and exhibit distinct physiological states, such as reduced metabolic activity and enhanced tolerance mechanisms, which make them significantly more resistant to antimicrobials compared to their planktonic counterparts. Consequently, MIC and MBC determinations do not accurately reflect the effectiveness of interventions against biofilm-embedded pathogens [62], 63].

To overcome these limitations, the minimum biofilm eradication concentration (MBEC) assay has been developed. This assay determines the concentration of an antimicrobial or treatment required to achieve a 99.9 % reduction of cells residing within a biofilm [62], [63], [64], [65], [66], [67], [68]. The MBEC assay provides a more physiologically relevant assessment of biofilm susceptibility, especially for pathogens commonly forming biofilms in the upper respiratory tract, such as *S*. *aureus*, *M*. *catarrhalis*, *H*. *influenzae*, and *C.* spp. Studies using the Calgary Biofilm Device have confirmed that biofilm-associated *S. aureus* exhibits increased antibiotic tolerance, highlighting the limitations of conventional therapies [69], 70]. Similarly, the MBEC assay has been valuable for evaluating antifungal treatments against *Candida* biofilms, which pose significant therapeutic challenges due to their resilience and structural complexity [71], [72], [73], [74]. More recently, the MBEC approach has been extended beyond conventional antimicrobials to assess non-pharmacological treatments, including hypertonic saline and salsobromoiodic thermal water solutions. These applications allow the study of alternative strategies for disrupting biofilms in a controlled, reproducible manner, providing insights into treatments that can target sessile microbial communities rather than planktonic cells, and thereby better reflecting clinical relevance in chronic upper airway infections [67].

In this study, we tested Rino Term^®^, a 2.5 % hypertonic salsobromoiodic thermal solution, for its antibiofilm efficacy. As reported in Table 1, its high sodium (9,376 mg/L) and chloride (15,094 mg/L) concentrations generate a strong osmotic gradient – approximately threefold higher than that of 0.9 % NaCl – which may promote cellular dehydration and disruption of the biofilm matrix. This mechanism is consistent with previous findings showing that hypertonic environments and ionic shifts can alter the structural stability and antibiotic susceptibility of bacterial biofilms [34], [75], [76], [77], [78], [79].

Beyond hypertonicity, the solution’s mineral composition may further contribute to its antibiofilm activity. Iodides (77.5 mg/L) may play a role through the generation of reactive iodine species, which are known to disrupt microbial membranes and oxidize essential cellular components, potentially affecting also biofilm-associated cells [80], [81], [82]. Bromides (143.0 mg/L), although not directly antimicrobial, have been associated with immunomodulatory effects and enhancement of mucosal defenses in thermal water therapies, which may indirectly support biofilm clearance [40], 83], 84]. Bicarbonate ions (122.0 mg/L) contribute to pH regulation and mucociliary clearance, thereby potentially limiting biofilm persistence [85], 86]. Moreover, divalent cations such as calcium (683 mg/L) and magnesium (327 mg/L), although generally involved in biofilm stabilization, may destabilize the extracellular polymeric matrix at high concentrations through competitive ionic interactions [87].

Overall, these physicochemical features – hypertonicity, halogen content, bicarbonate buffering, and mineral interactions – support a multifactorial model in which individual components may act additively or synergistically to interfere with biofilm formation and maintenance, in line with indirect evidence reported in the literature [84], [87], [88], [89], [90].

This study underscores the efficacy of hypertonic solution salsobromoiodic thermal water in disintegrating biofilms formed by bacterial and yeast pathogens associated with upper airway infections. Notably, hypertonic solution salsobromoiodic thermal water demonstrated a superior capacity to disrupt biofilms compared to saline solutions across all tested biofilms, including polymicrobial ones. Key results from washing experiments reflect the combined effects of the test solution and mechanical disruption by sonication. While treatment contributed to biofilm detachment, the current data do not allow quantification of the purely chemical effect. Sonication is widely recognized as an effective method for dislodging adherent biofilms and obtaining reliable quantitative measurements [91], 92]. Future studies should assess the effect of the test solutions without sonication to isolate their purely chemical action.

Acknowledging the persistence and adhesion of microbial biofilms, additional tests involved rigorous mechanical stress (sonication) at two-time points to provide deeper insights. The MBEC results confirmed that solutions containing hypertonic solution salsobromoiodic thermal water significantly reduced biofilm at both the first and second sonication steps compared to untreated controls. However, saline treatments alone failed to sustain this effect during the second sonication step for *C. albicans* DSM 1386 (Figure 10B). This outcome suggests that either the first sonication step alone could detach all biofilm for these strains or that the observed effects at shorter times were transient.

When comparing treatments, more concentrated hypertonic solution salsobromoiodic thermal water formulations consistently exhibited superior biofilm reduction compared to saline. For bacterial strains, biofilm reduction was closely linked to the concentration of thermal water, with treatments 1 and 2 showing the most pronounced effects. For *S. aureus* DSM 20231 and DSM 21705, the hypertonic solution significantly disrupted biofilm mass, aligning with previous studies demonstrating the role of hypertonic environments in destabilizing the extracellular biofilm matrix and enhancing bacterial susceptibility to antibiotics with a reduction % between 46.939 and 58.757 % (Figures 4 and 5 and Tables 3 and 4).

For *M. catarrhalis* DSM 9143 and DSM 11994, hypertonic solution salsobromoiodic thermal water treatment markedly reduced biofilm integrity with a reduction % between 32.467 and 53.279 % (Figures 6 and 7 and Tables 5 and 6). These findings highlight potential clinical applications for addressing biofilm-mediated chronicity and antibiotic resistance in respiratory infections caused by this pathogen. Similarly, the hypertonic solution effectively disrupted *H. influenzae* DSM 10001 biofilms (Figure 8 and Table 7) with a reduction % between 39.603 and 48.980 %, reinforcing its therapeutic potential for infections involving this biofilm-forming pathogen. *S. pneumoniae* DSM 14377 biofilms also exhibited substantial reductions in mass with a reduction % between 52.980 and 55.725 %, underscoring the solution’s relevance in combating biofilm-associated conditions such as otitis media and sinusitis (Figure 9 and Table 8).

Treatments applied to fungal pathogens, including *C. albicans* DSM 1386, *C. parapsilosis* DSM 4237 and *C. tropicalis* DSM 4238, showed less marked effects than those observed in bacteria, although the results obtained were statistically significant (Figures 10–12; Tables 9–11). In particular, the hypertonic bromo-iodine thermal water solution resulted in a reduction of *C. albicans* DSM 1386 biofilm by 23.404 % (Figure 10B, Table 9), a less pronounced effect than that observed in bacterial biofilms. Nevertheless, these results retain clinical relevance, given the high resilience of *Candida*-associated biofilms. Polymicrobial biofilms exhibited greater structural resilience compared to monospecific ones, requiring a longer sonication time (30 min) to achieve a significant reduction in biomass. All tested treatments showed comparable antibiofilm efficacy; however, the concentrated hypertonic salsobromoiodic thermal water solution (T1) produced a slightly higher reduction (∼51 %), confirming the increased resilience and complexity of polymicrobial communities compared to single-species biofilms (Figure 13, Table 12). Overall, the comparison among the tested species confirmed a clear differential susceptibility to the hypertonic salsobromoiodic thermal solution. Bacterial biofilms showed the most pronounced reductions, with decreases ranging from approximately 32–58 % depending on the strain, including the marked sensitivity of *S. aureus* DSM 20231 and DSM 21705 (46.9–58.7 %). In contrast, fungal biofilms exhibited a more limited response, with *C.* spp. showing reductions generally below 25 %, such as the 23.4 % decrease observed for *C. albicans* DSM 1386. These findings reflect the known resilience of *Candida* biofilms compared to bacterial ones and support a concentration-dependent effect of the hypertonic solution across different microbial communities.

The findings from this study confirm the broad-spectrum efficacy of hypertonic solution salsobromoiodic thermal water against Gram-positive and Gram-negative bacteria and fungal biofilm species. Its physiochemical properties support its therapeutic potential in chronic rhinosinusitis and similar conditions. Minerals like magnesium, calcium, potassium, and bicarbonate confers additional benefits and clinical studies further support the safety of hypertonic thermal saline [93], [94], [95], [96].

Despite these promising results, the limitations of this study must be acknowledged. The *in vitro* design cannot fully reproduce the complexity of the human respiratory tract, where host immune responses, epithelial interactions, and microbial dynamics play a crucial role in biofilm formation and treatment outcomes [97], [98], [99]. Regarding the polymicrobial biofilm, faithfully modeling clinical multispecies biofilms remains highly challenging. Variations in species composition, relative abundance, spatial architecture, and extracellular matrix organization differ substantially between infection sites and patients [10], [11], [12]. For this reason, a standardized 1:1 inoculum ratio was adopted in the present study to ensure experimental reproducibility and comparability, rather than to replicate the heterogeneous species proportions observed in clinical biofilms. Local microenvironmental factors such as pH, oxygen tension, and nutrient availability further influence biofilm structure and function [13], 15]. Moreover, host factors, including immune responses and epithelial interactions, cannot be reproduced *in vitro*, and interspecies interactions within multispecies biofilms can modulate metabolism, structural integrity, and antimicrobial tolerance [19], [20], [21]. *In vivo* experiments would provide crucial insights into these aspects by allowing the evaluation of treatment efficacy in the context of host-pathogen interactions, immune modulation, mucociliary clearance, and tissue repair. Such studies could clarify whether the observed antibiofilm effects translate into clinically meaningful outcomes, including reductions in infection severity, inflammation, and tissue damage. Additionally, *in vivo* models would enable the assessment of safety, tolerability, and potential cytotoxicity in a physiological environment, which cannot be fully captured *in vitro* [97].

In addition, the kinetics of polymicrobial biofilm formation were not monitored over time. Biofilms were allowed to develop for 48 h under standardized conditions to ensure the formation of mature and stable biofilm structures prior to treatment, in line with the primary aim of this study, which was to evaluate the antibiofilm activity of the hypertonic salsobromoiodic solution on established biofilms rather than to characterize early biofilm development. While the temporal dynamics of biofilm formation, including initial adhesion and microbial succession, were not assessed, these aspects remain of considerable interest and are recognized as a limitation of the present *in vitro* design. Future studies incorporating time-course analyses and species-specific methods, such as qPCR or fluorescence *in situ* hybridization (FISH), will be required to better characterize biofilm maturation kinetics and microbial succession within polymicrobial communities [100]. In addition, the present study was not designed to assess the effects of the hypertonic salsobromoiodic solution on planktonic microbial cells, including potential modulation of virulence factor expression. This choice reflects the specific aim of the study, which was focused on the evaluation of antibiofilm activity against mature mono- and polymicrobial biofilms. Nevertheless, previous studies have reported that hypertonic and saline-based formulations may influence microbial physiology by altering osmotic balance, reducing surface adherence, and interfering with quorum sensing and EPS production [101]. Such mechanisms could indirectly affect virulence-associated traits, particularly in pathogens such as *S. aureus* and *C. albicans* [102]. These observations are provided here for contextual purposes only and were not directly investigated in the present work.

Similarly, potential synergistic interactions between the tested solution and conventional antimicrobial agents were not explored within the scope of the current experimental design. While several studies have suggested that hypertonic or iodine-containing solutions may enhance antibiotic or antifungal efficacy by increasing membrane permeability or reducing biofilm tolerance [77], 78], 82], this study does not address such interactions. Future investigations specifically designed to evaluate combination therapies will be necessary to determine whether the hypertonic salsobromoiodic solution could potentiate standard antimicrobial treatments against biofilm-associated pathogens. A limitation of this study is that biofilm quantification after treatment was performed following mechanical disruption by sonication. Because the tested formulation is a hypertonic solution salsobromoiodic thermal water rather than a classical antimicrobial agent, the experimental approach was designed to evaluate antibiofilm efficacy under conditions combining chemical exposure with mechanical stress, a strategy commonly adopted *in vitro* biofilm models to enable reproducible detachment and quantitative recovery of adherent cells. Sonication is widely used in biofilm research and in the diagnosis of device-associated infections to disrupt the biofilm matrix and improve bacterial recovery [103], [104], [105], [106]. However, sonication generates physical perturbations such as cavitation and pressure waves that do not directly reproduce physiological hydrodynamic shear forces [103], 106]. Accordingly, the results presented here reflect the combined effect of chemical treatment and mechanical disruption. The absence of measurements obtained prior to sonication represents a limitation, and future studies should include parallel experiments without mechanical disruption to better isolate the purely chemical effects of hypertonic solutions on biofilm stability.
